# 3D retinal imaging and measurement using light field technology

**DOI:** 10.1117/1.JBO.26.12.126002

**Published:** 2021-12-17

**Authors:** Stefan Schramm, Alexander Dietzel, Dietmar Link, Maren-Christina Blum, Sascha Klee

**Affiliations:** a Technische Universität Ilmenau, Institute of Biomedical Engineering and Informatics, Faculty of Computer Sciences and Automation, Ilmenau, Germany; b Karl Landsteiner University of Health Sciences, Division Biostatistics and Data Science, Department of General Health Studies, Krems, Austria

**Keywords:** light field, fundus imaging, glaucoma, eye model

## Abstract

**Significance:** Light-field fundus photography has the potential to be a new milestone in ophthalmology. Up-to-date publications show only unsatisfactory image quality, preventing the use of depth measurements. We show that good image quality and, consequently, reliable depth measurements are possible, and we investigate the current challenges of this novel technology.

**Aim:** We investigated whether light field (LF) imaging of the retina provides depth information, on which structures the depth is estimated, which illumination wavelength should be used, whether deeper layers are measurable, and what kinds of artifacts occur.

**Approach:** The technical setup, a mydriatic fundus camera with an LF imager, and depth estimation were validated by an eye model and *in vivo* measurements of three healthy subjects and three subjects with suspected glaucoma. Comparisons between subjects and the corresponding optical coherence tomography (OCT) measurements were used for verification of the depth estimation.

**Results:** This LF setup allowed for three-dimensional one-shot imaging and depth estimation of the optic disc with green light. In addition, a linear relationship was found between the depth estimates of the OCT and those of the setup developed here. This result is supported by the eye model study. Deeper layers were not measurable.

**Conclusions:** If image artifacts can be handled, LF technology has the potential to help diagnose and monitor glaucoma risk at an early stage through a rapid, cost-effective one-shot technology.

## 1 Introduction

Fundus photography is an established method for the diagnosis and documentation of retinal diseases.^1^ One approach to imaging three-dimensional (3D) structures, such as the optic nerve head, is stereoscopic fundus photography, which generates a stereo image using two fundus images from slightly different viewing angles. This image gives a three-dimensional impression when viewed through stereo glasses. However, depth measurement and depth information are not provided.^2^^–^^4^

Current developments in the field of fundus imaging are dominated by scanning laser technologies that do not have the advantage of one-shot technology but have a higher diagnostic potential^5^^,^^6^ than classical fundus photography. However, with the novel development of 3D light field (LF) photography,^7^^–^^10^ fundus photography can be expanded in terms of depth information. This increases the diagnostic possibilities; facilitates photography by digitally refocusing or enlarging the depth of focus to a maximum, resulting in a total focus image-after-image capture; and improves image quality.^11^ In particular, the 3D imaging of the optic nerve head and the quantitative analysis of the excavation can be applied in glaucoma diagnostics.^3^^,^^4^ One strong risk factor for glaucoma is a suspicious optic nerve head appearance with abnormal cupping or an increase in the cup-to-disc ratio.^12^^,^^13^ With demographic changes in industrialized countries, the incidence of glaucoma is increasing.^14^^,^^15^ As this eye disease is one of the most common causes of blindness,^16^^,^^17^ cost-effective, rapid, and precise screening is a key factor in rapid medical intervention.

LF technology has the potential to be less expensive since no internal moving parts are needed, and the one-shot principle facilitates faster imaging compared with conventional scanning technologies. The latter are in need of intelligent compensation mechanisms for eye movements during the imaging process.

The first applications of fundus imaging utilizing LF technology were addressed by some patents^18^^–^^20^ and publications.^11^^,^^21^ Palmer et al.^11^ showed that image enhancement regarding suppression of glare and depth mapping of the fundus are possible. Thurin et al. also developed an LF fundus camera for 3D imaging.^21^ Both showed the possibility of retinal LF imaging in principle, though image quality seemed to be much lower than in standard fundus photography. From their example images, no diagnostic parameters were determined.

Marshall et al. investigated the depth estimation of the ocular fundus with LF technology. The authors postulated that, because there is not enough image structure in a typical retinal image, no useful depth estimation is possible.^22^ Furthermore, it was demonstrated that depth estimation of deeper fundus layers is not possible. In theoretical examinations, they showed that scattering in the overlying layers destroys the directional information of single rays.^23^

What was common to all approaches documented in the literature was that the LF fundus image quality was very limited. One reason for the poor image quality compared with conventional fundus imaging was the optical setups, which are optically designed and constructed very simply compared with typical fundus camera optics.

The goal of this study is to demonstrate depth estimation at the human fundus by one-shot imaging. Therefore, a standard fundus camera providing a well-designed optical system is equipped with an LF imager adapted to the area of the optic disc. In contrast to the known approaches from the literature, a basic requirement is an adequate physical eye model. This model addresses the challenges of illumination and imaging of the human eye and includes a corresponding depth extension of the optic nerve head. With this approach, we aim to answer the following fundamental questions.

(1) Is depth at the ocular fundus at all measurable with an LF fundus camera?
(2) On which structures is the depth estimated?
(3) What wavelength produces the most depth information?
(4) Are structures at deeper layers, such as the choroidea, measurable?
(5) What kinds of image artifacts occur?

## 2 Methods

### 2.1 Subjects

We examined six eyes (three right and three left eyes) of three healthy subjects (aged 22, 24, and 30 years) and three subjects with suspected glaucoma (aged 32, 37, and 46 years). The latter three subjects were known to have a conspicuous optic disc, their intraocular pressure was within the normal range, and there were no visual field defects. All subjects gave their written informed consent in accordance with the declaration of Helsinki. To minimize the influence of light scatter and wavefront errors, the exclusion criteria were dry eye syndrome, cornea defects, edema, and cataract. Refractive errors were limited to −2 to +1 dpt spherical and −0.5 to +0.5 dpt cylindrical. All subjects underwent a preliminary examination to rule out eye diseases.

### 2.2 Eye Model

**Fig. 1:**
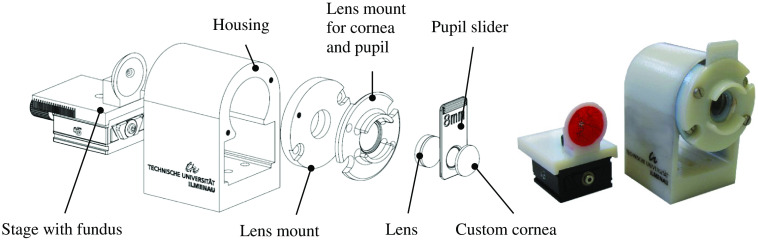
Exploded view of the eye model and photography of the fundus model and the eye model; the components from left to right: changeable fundus on a miniature dovetail translation stage, housing, lens mount for the lens, lens mount for cornea and pupil, lens, changeable pupil slider (here, an 8-mm pupil diameter), and custom cornea.

To validate the depth estimation and to show its possibilities and limitations, an eye model (scale 1:1) incorporating an interchangeable fundus was designed and realized (Fig. 1). The scale was chosen to realistically mimic the geometry of the human eye, especially the anterior parts starting with the cornea. This is a crucial factor in terms of strong reflections and disturbing stray light by the interaction of illumination and imaging light. To avoid low-contrast images due to overlaying reflections and stray light in the imaging path, the separation of illumination and imaging at these parts of the eye is necessary.

**Fig. 2:**
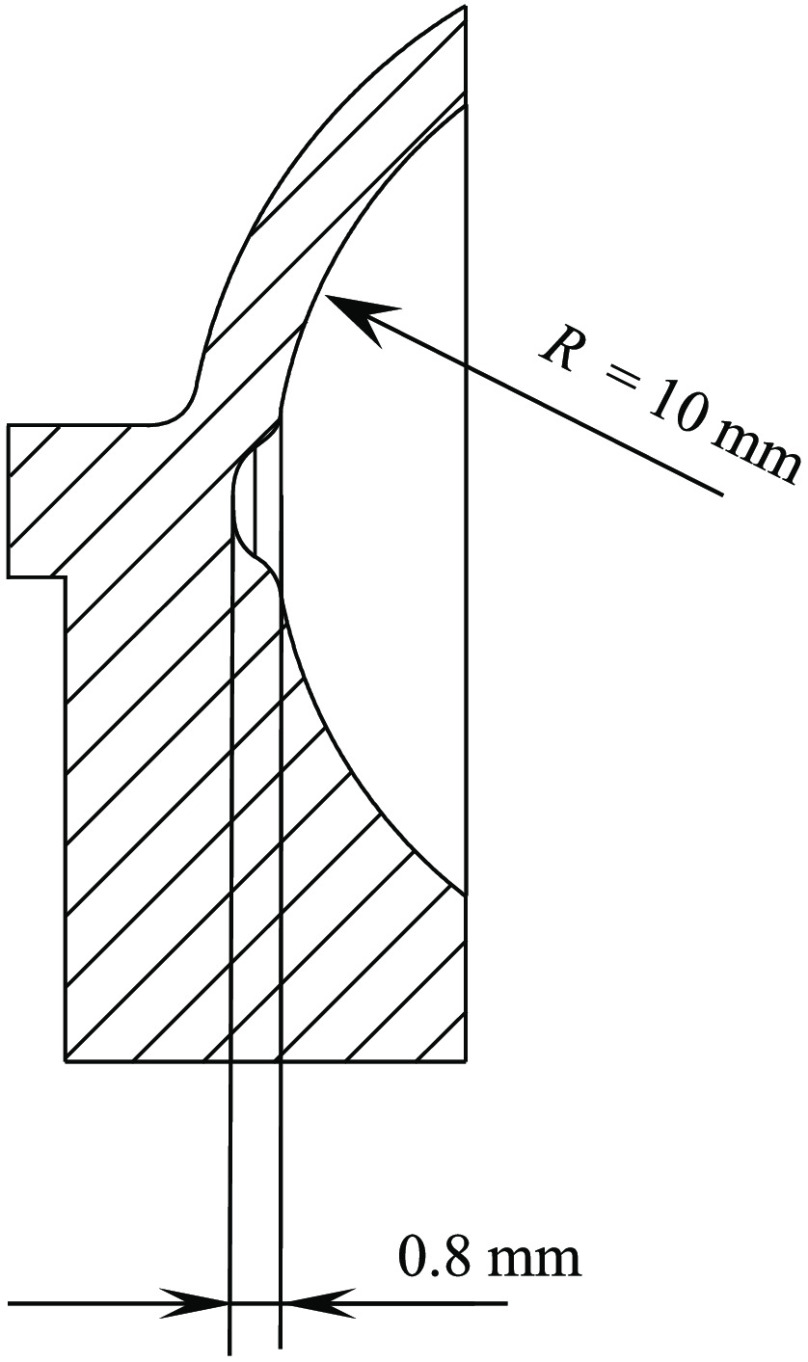
Cross section of the fundus model with a fundus radius of 10 mm and a papilla depth of 0.8 mm, as an example.

The fundus had a radius of 10 mm and papilla excavations of 0.2, 0.4, 0.8, and 1.0 mm, respectively (Fig. 2). The eye model’s total length was 24.25 mm. The cornea was a custom, uncoated N-BK7 lens (outer radius 7.707 mm, inner radius 8.886 mm, and thickness 1.5 mm). The lens was an uncoated achromat (AC127-19, Thorlabs GmbH, Munich, Germany). The lens mount, housing, and fundus were printed with a 3D printer (Objet30 Prime, Stratasys Ltd., Eden Prairie, USA) with a resolution of 25  μm. The material used was white plastic (VeroWhite™, Stratasys Ltd., Eden Prairie, USA). The fundus models were also painted with red acrylic lacquer (Molotow™, Feuerstein GmbH, Lahr, Germany), and vessels were drawn with a thin brush by a professional artist.

### 2.3 Technical Setup (LF Imaging Device)

**Fig. 3:**
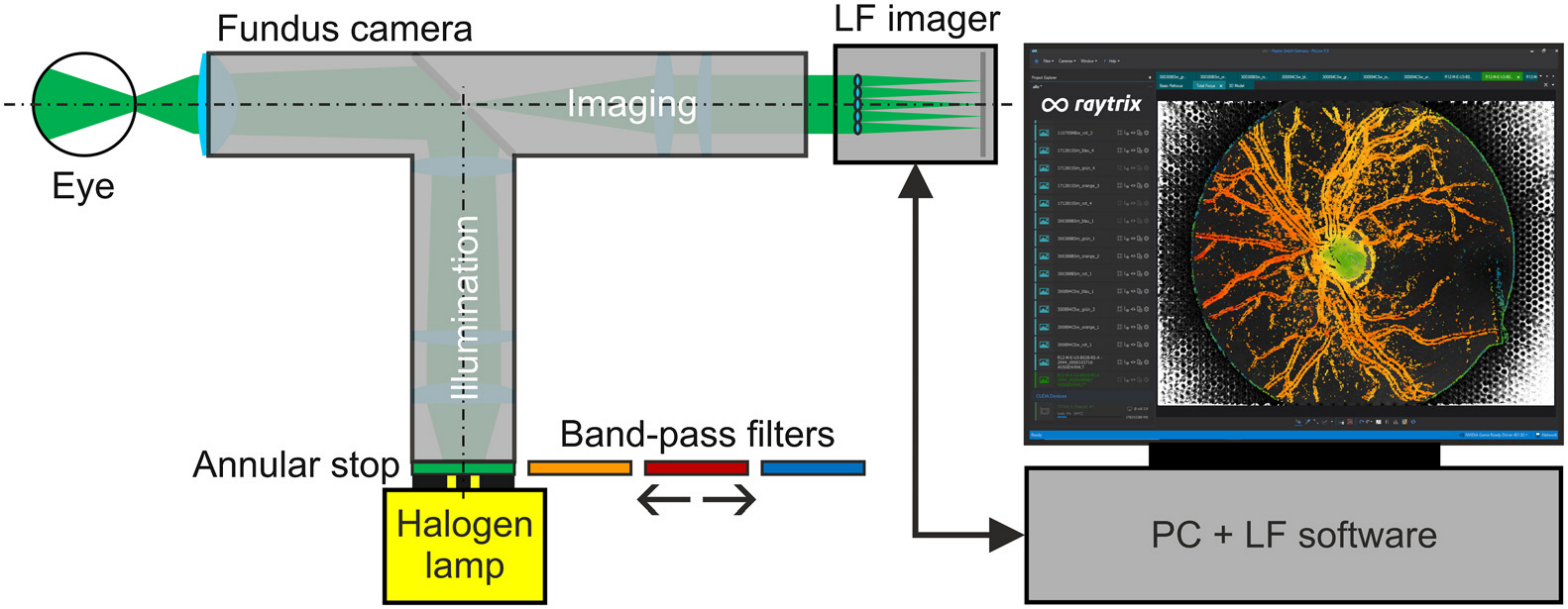
Technical setup consisting of a fundus camera with an adapted LF imager which is operated with the Raytrix LF software (RxLive 5.0.046.0, Raytrix GmbH, Kiel Germany). The fundus camera is equipped with a broadband halogen lamp, while four narrow bands are realized by means of bandpass filters (450±20,520±20,600±20, and 650±20  nm).

The technical setup, shown in Fig. 3, consisted of a mydriatic fundus camera (FF450, Carl Zeiss Meditec AG, Jena, Germany) and an LF imager (R12, Raytrix GmbH, Kiel, Germany) connected via a c-mount adapter. The LF imager was operated with the Raytrix LF software (RxLive 5.0.046.0, Raytrix GmbH, Kiel, Germany).

The LF imager consisted of a Basler industrial camera (type: acA4024-29um, Baser AG, Ahrensburg, Germany) and a microlens array. This camera had a 4024×3036  pixel monochrome 16 bit CMOS sensor (type: Sony IMX226CLJ-C) with an effective sensor diagonal of 9.33 mm, a pixel size of 1.85×1.85  μm, and a maximum frame rate of 29 frames per second. The microlens array had lenses with a diameter of 80  μm in a hexagonal grid of three focal lengths to increase the imageable depth.^9^^,^^10^

The setup corresponds to a focused plenoptic camera, sampling the LF of an intermediate image with microsubimages, i.e., the microlenses project small subimages of the scene onto the sensor. Each subimage shows a slightly different view. If an object point can be detected in at least two subimages, a virtual depth can be estimated similar to stereo photography approaches. The detection of two corresponding object points is done by autocorrelation of small pixel patches, which require a minimum level of contrast of image structure. Parameters for adapting this autocorrelation function are given in the Appendix. If the corresponding object points are known, a triangulation is performed and the disparity or virtual depth is determined. A detailed description of the algorithmic image reconstruction and depth estimation is given by Perwaß and Wietzke.^10^

The use of a focused plenoptic camera enabled image reconstruction with high spatial resolution in contrast to a conventional LF camera, the lateral resolution of which is limited by the number of microlenses.^7^^,^^8^ To achieve the maximum angular resolution of the LF imaging and thus make use of the maximum depth resolution, the f number of the preceding optic (here, the optic of the fundus camera) should be equal to the LF camera.^8^^–^^10^ The LF camera has an f number of 2.4. To best match the optical aperture of the fundus camera, the field of view of the fundus camera optics was set to 30 deg (20 deg, 30 deg, and 50 deg were possible). With the resulting lateral magnification of 1.5 and 2.25 in the direction of the optical axis, the depth resolution of the technical setup can be estimated at 18  μm.

To perform spectral depth measurements, four bandpass filters (Thorlabs GmbH, Munich, Germany) with central wavelength ± bandwidth (full-width at half-maximum) values of 450±20  nm (blue, FB450-40), 520±20  nm (green, FBH520-40), 600±20  nm (orange, FB600-40), and 650±20  nm (red, FB650-40), respectively, were inserted into the illumination path of the fundus camera optics using customized sliding filter mounts. The eye model was illuminated with green light, with a wavelength of 520±20  nm.

### 2.4 Optical System Characterization

To determine the optical performance of the overall system, the modulation transfer function (MTF) at the optical axis was determined and compared with the MTF of the same optical system utilizing a conventional CCD imager instead of the LF imager. For this purpose, a razor blade edge was positioned vertically in the center of the fundus plane of the eye model. It was homogeneously back-illuminated with green light (520 nm). A total focus LF image and a conventional image using a CCD camera (Stingray F-046, 780×560  pixels, 8.3×8.3  μm, Sensor Sony ICX415, Allied Vision, Stadtroda, Germany) were taken. The MTF curves were then calculated using MATLAB (R2018b, MathWorks) by edge differentiation of the average edge profile of 50 rows each with a length of 400 pixels, with a subsequent Fourier transform.^24^

### 2.5 Imaging Procedure

The imaging procedure and analysis are shown in Fig. 5. Each subject underwent an examination via optical coherence tomography (OCT Spectralis, Heidelberg Engineering GmbH, Heidelberg, Germany) with the glaucoma module (Glaucoma Module Premium Edition, Software Version 6.0, Heidelberg Engineering GmbH, Heidelberg, Germany) using the ONH-RC scan pattern (optic nerve head-radial circle) (OCT-scan, Fig. 5). After detecting and confirming the fovea position, the center of the basal membrane opening (BMO) was detected, adjusted if necessary, and confirmed. The c-curve (meaning corneal radius) was individually measured via an autorefractor (CX 1000, Rodenstock GmbH, Munich, Germany) and entered. The acquiring process included 24 radial scans centered above the BMO with a rotation distance of 7.5 deg and three circle scans (3.5/4.1/4.7-mm-diameter). For the radial scans, 25 continuous scans were cumulated, whereas 100 continuous scans were cumulated for the circle scans. Subsequently, the same eye of each subject was set under mydriasis (Mydriaticum Stulln, Pharma Stulln GmbH, Stulln, Germany). After a period of 15 min for pupil dilatation, LF images were taken (LF image acquisition, Fig. 5).

For each illumination bandpass filter, three images were captured, and the filter sequence was randomized. For illumination, the standard light source of the fundus camera was used (Fig. 3). The illumination intensity was individually set as bright as was acceptable to each subject. The shutter time and gain of the LF imager were set close to saturation, avoiding overexposing the papilla region. In the eye-model study, 30 images were taken for each papilla depth. For each image, the LF fundus camera was readjusted, including the illumination intensity and exposure parameters of the LF imager.

### 2.6 Image and Measurement Analysis

For OCT-depth measurement, 2 of the 24 radial OCT scans were selected with an excellent visible base of the optic cup and prominent vessel structure above (or slightly behind) the neuroretinal rim of the papilla for each subject (OCT-depth measurement Fig. 5). One of the selected scans was more vertically oriented, whereas the other was more horizontally oriented; this depended strongly on the individual vessel tree of each subject. In the analysis, only horizontal and vertical scans were used. In each selected scan, the lowermost base position equal to the deepest spot in the optic cup and the uppermost vessel position equal to the uppermost position of the optic disc were marked graphically by horizontal lines; the distance between these two lines, equal to the depth, was measured with a vertical line (Spectralis, Glaucoma Module Premium Edition, Software Version 6.0, Heidelberg Engineering GmbH, Heidelberg, Germany) (Fig. 15). This measurement process was done with the help of the overlay tool and its option to measure distances in μm. Both results were averaged.

**Fig. 4:**
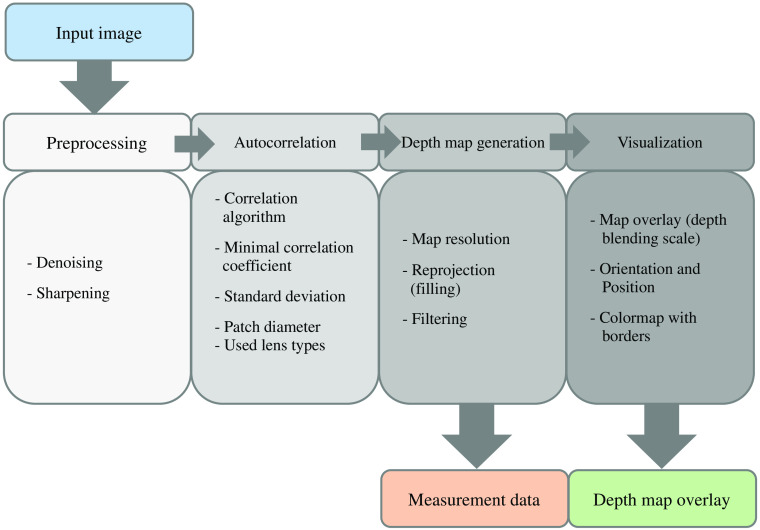
Image processing chain of the LF image analysis using Raytrix LF software (RxLive 5.0.046.0, Raytrix GmbH, Kiel, Germany). The input image is first preprocessed (not used here); subimages are autocorrelated, triangulated, and depth estimated; a depth map is generated in which the papilla depth was measured; and finally the depth-map is visualized.

For LF image analysis, Raytrix LF software (RxLive 5.0.046.0, Raytrix GmbH, Kiel, Germany) was used. Detailed software parameters and settings used can be found in the Appendix. The image processing procedure within the Raytrix LF software is shown in Fig. 4. First, the input image passes preprocessing, which was disabled here to not change the image data. In a second step, subimage autocorrelation and triangulation for depth estimation were performed. The third step generated a depth map, and data were filtered conservatively here. In the last processing step, the corresponding depth map was visualized.

**Fig. 5:**
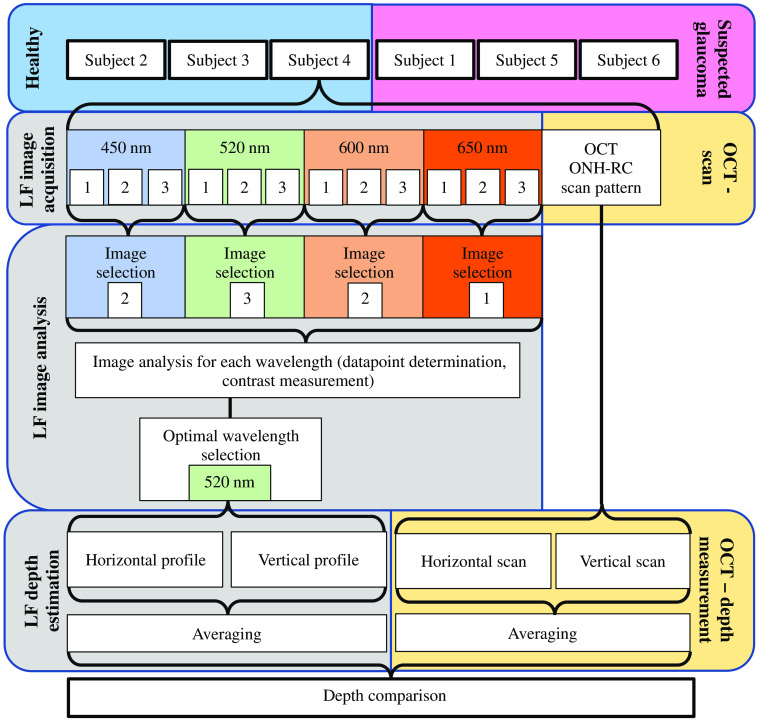
Flowchart of the subject study using subject 4 as an example.

For the analysis of the study data, the image quality of the LF images was first subjectively proved regarding sharpness, motion blur, and artifacts. The best of three images was taken for further analysis (LF image analysis, image selection, Fig. 5).

The quality of the fundus images regarding depth reconstruction at different wavelengths was evaluated by analyzing the Michelson contrast and by counting the number of reconstructed depth positions using MATLAB (R2018b, MathWorks). This evaluation was restricted to a region of interest (ROI) due to the presence of image artifacts with varying intensity and manifestation, different occurrences of the fixation needle, the influence of the aperture diaphragm, and a change in visibility of structures. To ensure a comparable evaluation across all images, the center of the ROI needed to correspond with the center of the papilla, and a circular region was defined. To assure minimal differences between the center positions, the more prominent rim was marked in clockwise order for each image: north, east, south, and west. The horizontal center of the papilla was calculated by dividing the distance between the east and west coordinates, whereas the vertical center used north and south.

Furthermore, the horizontal and vertical extensions of the papilla allowed for the calculation of the largest papilla radius with 185 pixels. The Michelson contrast was analyzed by selecting measurement positions in the total focus image along an imaginary ring at a distance of two times the papilla radius from its center at the three largest venules. Arterioles were not considered because of the limited visibility, especially at 650 nm. Three measurement positions were stored per venule, one above and the other two on opposite sides of the venule. Concerning the small but nearly visible bubble-like artifacts that increased the gray values locally, the measurement was taken with an eroded version of the total focus image using a disc-shaped structuring element with a radius of 8 pixels. This erosion operation worked like a filter and stored the lowest gray value inside the structuring element at its center. The three separate Michelson contrast values per image were averaged for a clear presentation.

As a second quality parameter, the number of reconstructed depth positions per image was counted inside a circular ROI with a diameter of 5.5 times the largest papilla radius of 185 pixels. The factor 5.5 represents the maximum possible ROI, avoiding the overlap of the aperture diaphragm while also keeping the ROI inside the image itself. Papilla depth was then determined at the wavelength with the most depth measurement points (LF image analysis, optimal wavelength selection, Fig. 5).

For this purpose, the position in the Z direction of the optic nerve head was determined on an average circle area of 20 pixels, including the ground of the optic nerve head. The position in the Z direction of the surrounding area was determined based on the two radial scans from the OCT and the selected measurement locations. Again, an averaged circle area of 20 pixels was used. Both measuring points were selected manually. The depth of the optic disc was determined from the difference between the optic disc periphery and its ground. This depth determination was performed according to the OCT scans in the horizontal and vertical directions. The resulting depths of the optic nerve head were averaged for each eye. The depth specifications correspond to a virtual estimation in virtual mm (v-mm) (LF-depth estimation, Fig. 5), which can be converted to mm with the calculated image scale of 2.25 in the Z direction.

The papilla depth of the eye model was determined on an averaged circle area of 85 pixels, including the complete ground of the optic nerve head. All 30 measurements for each papilla depth were averaged and compared with the printed depth. The imaging procedure and analysis are shown in Fig. 5. Six subjects were examined. Each subject was examined with four wavelengths for LF imaging and underwent an OCT examination. Three LF images per wavelength were taken, and one OCT scan (ONH-RC scan pattern) was conducted. Of the three LF images taken per wavelength, one was chosen for analysis. The four resulting LF images per subject were analyzed with regard to vessel contrast and depth data point found. The image with the highest vessel contrast and most datapoints was selected. Within this image, the papilla depth was estimated horizontally and vertically. Both data points were averaged. A corresponding depth measurement was performed in horizontal and vertical OCT slices. Both data points were averaged. Results of the LF estimation and OCT measurement were compared.

## 3 Results

### 3.1 MTF Determination

**Fig. 6:**
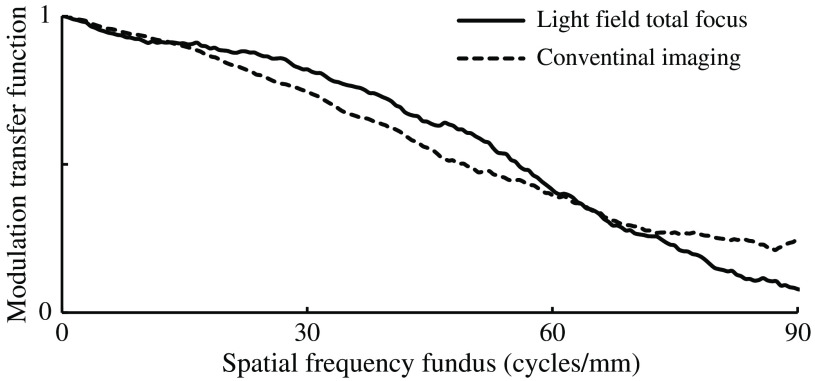
MTF curves of the overall system in object space (fundus of the model eye), using the LF setup in total focus mode (circles) and the system using a conventional CCD imager.

The minimum MTF amplitude for the LF imager occurred at ∼90  cycles/mm and was therefore chosen as the cutoff frequency. The MTF graph of the LF imager and the conventional camera have very similar progressions, whereas the MTF of the LF imager shows a slightly better performance in the middle with ∼56  cycles/mm at a relative amplitude of 0.5 in the object space compared with 45  cycles/mm for the conventional imaging (Fig. 6).

### 3.2 LF Imaging

**Fig. 7:**
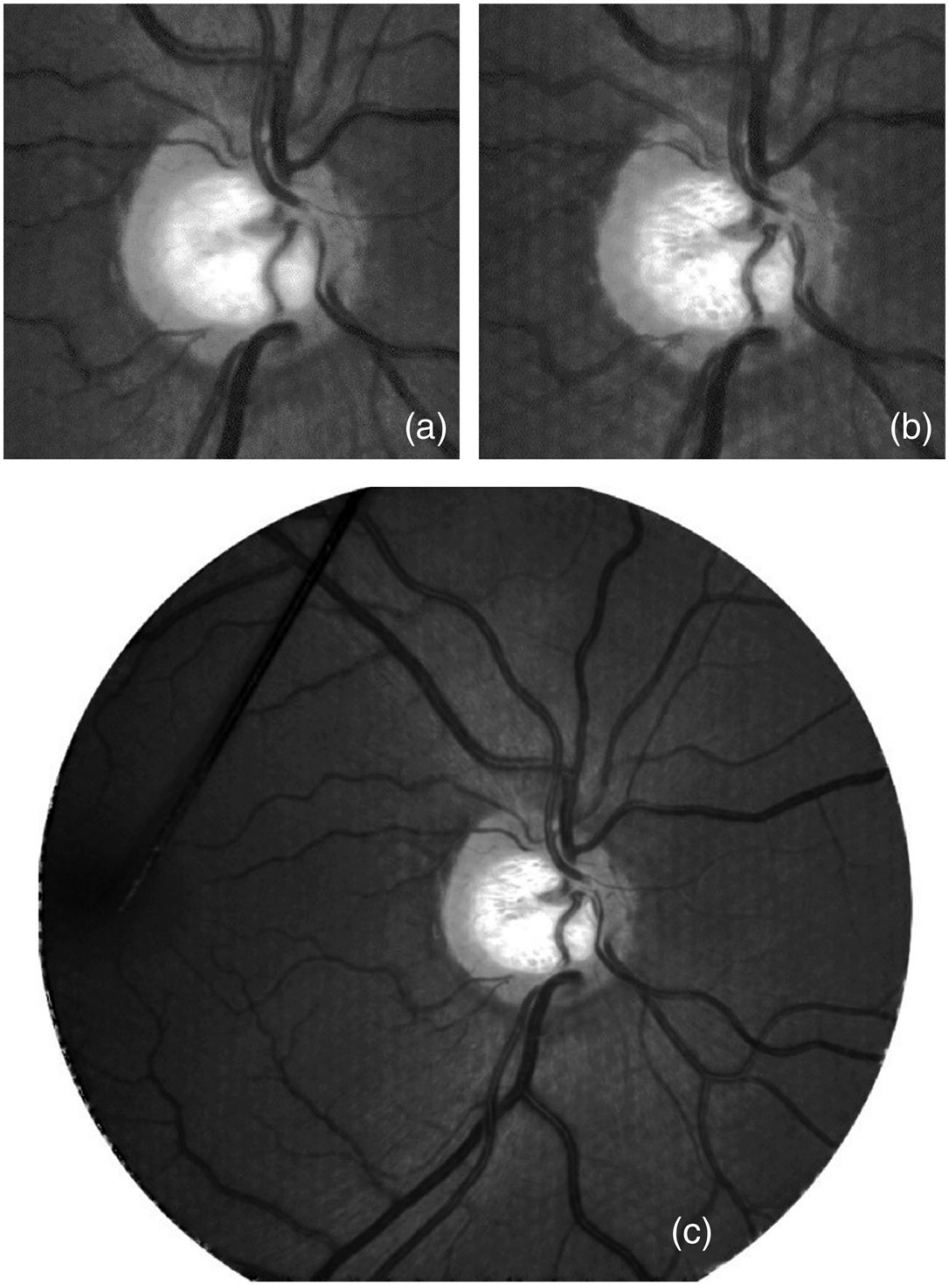
Refocused partial images (∼10-deg field of view) of a suspiciously glaucomatous papilla: (a) retina focused, (b) papilla bottom focused, and (c) total focus image with maximum depth of focus (full field).

It was possible to perform 3D fundus imaging in an eye model as well as *in vivo* by means of a novel LF-based optical setup. Subsequent digital refocus was possible in all LF images. Figure 7 shows examples of digitally refocused images of the papilla of subject S1 with suspected glaucoma. Figure 7(a) is focused on the retina and features sharp vessels and a blurred papilla bottom. Figure 7(b) is focused on the papilla bottom; the retinal structures are blurred, with circular artifacts in a hexagonal grid. Figure 7(c) shows the total focus image with the maximum depth of focus.

### 3.3 Eye Model Measurements

**Fig. 8:**
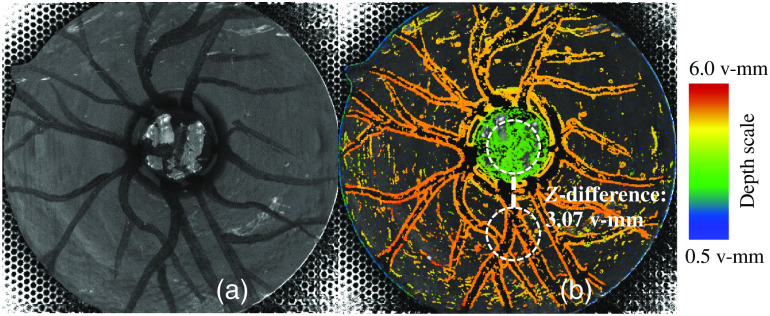
(a) Total focus image of the eye model with a papilla depth of 1.0 mm. (b) corresponding depth map with papilla cupping clearly visible; depth map showing only small gaps in gray; horizontal depth is measured as Z-difference between papilla center and retinal surface (dashed line, measurement area in dashed circles).

**Fig. 9:**
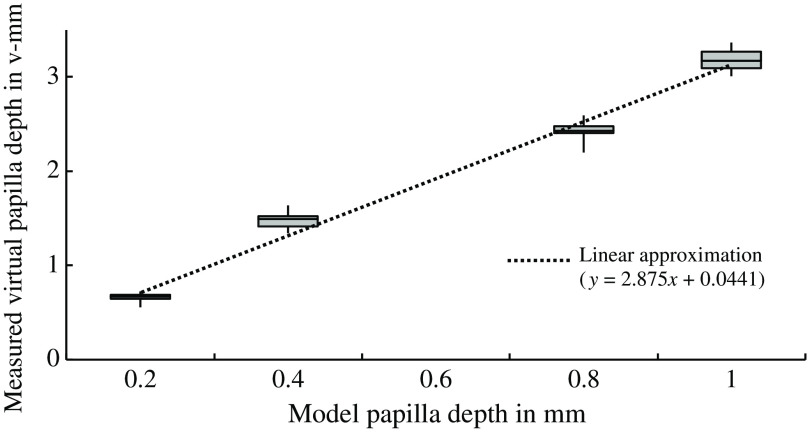
Relationship between the measured depth of the papilla in v-mm and the depth of the papilla in the eye model in mm. The boxplots represent the distribution of model papilla depths over n=30 LF measurements per model. Dotted line: linear fit with y = measured virtual papilla depth and x = model papilla depth.

Depth estimation of the eye model was possible over a 30-deg field of view (Fig. 8). Colored values represent the reconstructed depth estimation at the specific location. Depth estimation worked in regions with high-contrast structures, i.e., vessels and papilla. Papilla depths were measured from the middle of the papilla to the surrounding flat image region. Results are shown in Fig. 9. A linear relationship between the virtual depth and the model papilla depth can be assumed.

### 3.4 Spectral Imaging Versus Depth Estimation

**Fig. 10:**
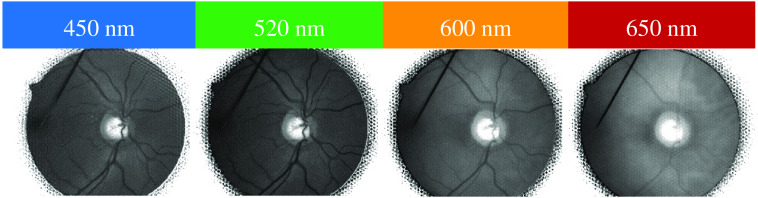
Total focus images of subject S1 for all wavelengths. From left to right: 450 nm (blue), 520 nm (green), 600 nm (orange), and 650 nm (red). Vessel contrast decreases with increasing wavelength.

**Fig. 11:**
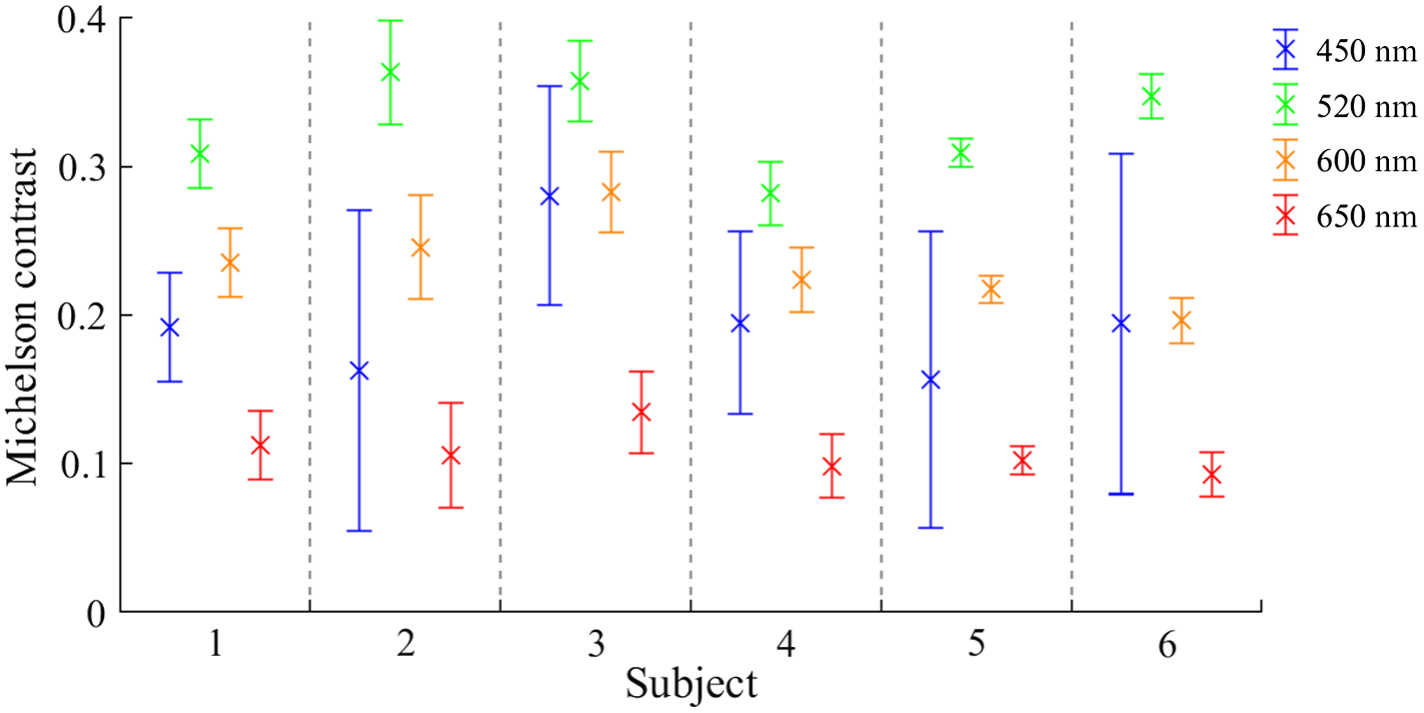
Averaged Michelson contrast and standard deviation of three venules per subject and wavelength: 450 nm (blue), 520 nm (green), 600 nm (orange), and 650 nm (red).

Figure 10 shows the LF images in total focus for subject S1. The vessel contrast is reduced at 600 and 650 nm. At 650 nm especially, veins are barely visible, whereas the choroidea becomes more prominent. The highest Michelson contrast per subject was found at wavelength 520 nm (see Fig. 11). The standard deviation was comparably low for all wavelengths, excluding 450 nm but including the favorable 520 nm.

**Fig. 12:**
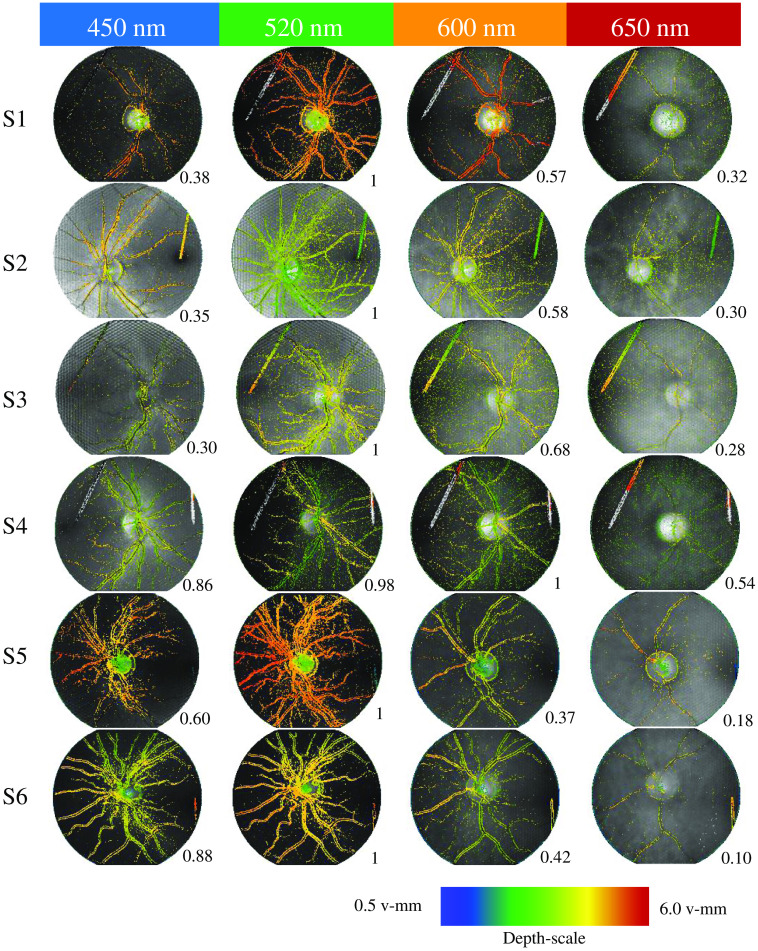
LF images of all subjects and wavelengths. Total focus images with the corresponding depth map overlays and measurement points normalized to the maximum per subject are shown. Most measurement points were found at a wavelength of 520 nm (for subjects S1 to S3, S5, and S6). Subject S4 had slightly more found measurement points at 600 nm. The internal fixation needle in the first intermediate image plane of the fundus camera (straight line within the fundus images) is imaged at S1 to S4 and S6 and depth-estimated thereon.

Figure 12 shows all total focus images with the corresponding depth map for all subjects and wavelengths. The maximum numbers of reconstructed depth positions per eye were as follows: 219,504 (S1), 295,941 (S2), 203,701 (S3), 146,922 (S4), 332,798 (S5), and 270,805 (S6). The normalized distribution per wavelength was included in this figure. The highest number of reconstructed depth positions per subject was typically at 520 nm. Only for subject S4 was the wavelength of 600 nm slightly better, with <2% more reconstructed depth positions. The other subjects showed a decreased number of values between 32% and 63%. The lowest number of reconstructed depth positions per subject was at 650 nm. Depth estimation only works in image areas with structures. Those structures are the retinal vessels, papilla border, and structures within the papilla. Choroidea was not measured.

**Fig. 13:**
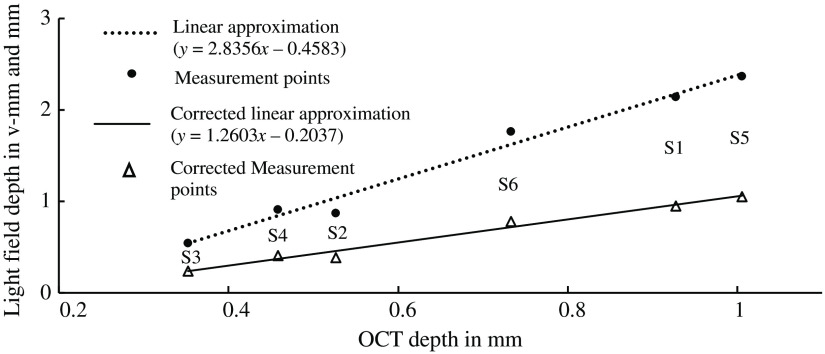
Comparison of LF papilla depth measurements with OCT data. Dots: papilla depth measurement with LF camera in v-mm compared with OCT measurements. Dotted line: linear fit with y = LF papilla depth and x = papilla depth measured with OCT; triangles: papilla depth measurement with LF camera corrected with the calculated magnification of depth of 2.25 in mm compared with OCT measurements, solid line: linear fit with y = corrected LF papilla depth and x = papilla depth measured with OCT.

Furthermore, the papilla depth was greatest for subject S5, corresponding to the deepest measured excavation with the OCT shown in Fig. 13. This subject has suspected glaucoma. The flattest papilla was observed in subject S2. The comparison of the OCT measurement and the LF depth estimation shows a linear relationship and an offset (Fig. 13).

**Fig. 14:**
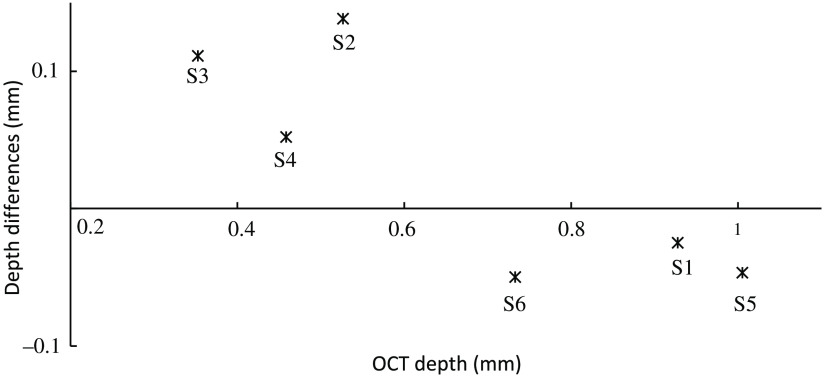
Depth differences of the magnification corrected LF depth estimation and the OCT measurements.

Figure 13 shows additionally corrected LF depth papilla measurements. The measurements were corrected with the calculated depth magnification of 2.25. They are in better agreement with the OCT measurements. Differences in the corrected LF papilla depth and the OCT measurements (Fig. 14) show that flat papillae are overestimated with a maximum measurement difference of 138  μm and deep papillae are slightly underestimated with a minimum measurement difference of −25  μm at subject S1.

**Fig. 15:**
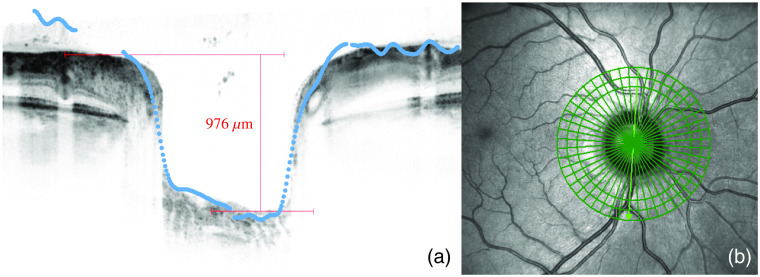
(a) Vertical papilla OCT scan of subject S1 with the vertical depth profile of the LF system (blue dots) and (b) SLO scan of the OCT showing the scan orientation.

Figure 15(a) shows a vertical papilla OCT scan of subject S1. The blue dots indicate the scaled vertical depth profile of the LF system. The excavation with a flat bottom and steep walls can be clearly seen in both systems.

### 3.5 Artifacts

**Fig. 16:**
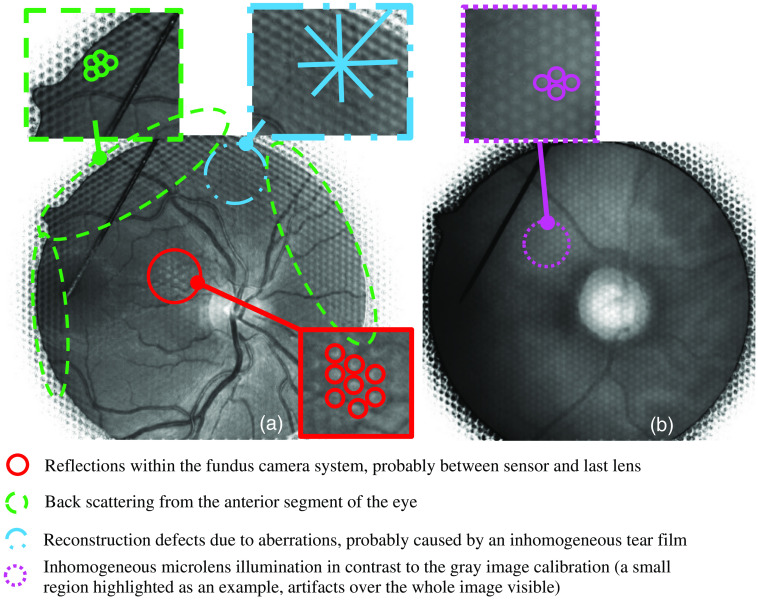
(a) Image artifacts at 520 nm (total focus, subject S3) and (b) image artifacts at 650 nm (total focus, subject S1).

For subject S3 especially, image acquisition was challenging due to unsteady fixation and small pupil despite mydriasis, resulting in significant image artifacts. The artifacts are visualized in Fig. 16. Reflections and scattering from the cornea and lens led to large checkerboard-like artifacts that cannot be subsequently removed. Internal system reflections were visible in all images of all subjects, especially with green light. Presumably, these reflections were generated between the last system lens or prism and microlens array [Fig. 16(a)]. All images taken with red light show round, blurred dots in a hexagonal grid [Fig. 16(b)].

## 4 Discussion

In this study, we answered fundamental questions about LF fundus imaging. The five questions presented in the introduction are addressed and discussed below.

(1) Is depth at the ocular fundus at all measurable with an LF fundus camera?

We showed that LF imaging of the human retina, especially the optic nerve head, is possible, and depths can be quantitatively determined. Image reconstruction produced subjectively high-contrast, good-resolution images compared with results of other authors working on LF fundus photography.^11^^,^^21^ The vascular contrast recorded with our LF system also corresponds to values in the literature measured with conventional systems.^25^^,^^26^ Compared with images from conventional fundus cameras, a detailed image from our system requires ideal conditions such as a large pupil and little stray light. Dilation of the pupil is also a basic requirement for a conventional mydriatic fundus camera. Here care should be taken to wait for maximum mydriasis. Stray light also reduces image quality in conventional fundus photography. In LF fundus photography, however, this leads to more severe artifacts, so patients with cataract benefit only to a limited extent from this examination method. However, approaches to compensate for stray light artifacts have already been published by other authors.^11^

The LF system shows a similar but slightly better MTF characteristic compared with the system using a conventional CCD imager, which is most likely due to its smaller pixels and thus higher resolution compared with Thurin et al., who measured about 10  cycles/mm at MTF=0.5; we achieved better results with about 56  cycles/mm.^21^ In comparison with Thurin et al., we additionally included the optics of the model eye for MTF determination to obtain a more realistic estimate. The depth differences of the optic nerve head of the subjects with suspected glaucoma compared with those of the healthy subjects are clearly visible and measurable (Fig. 13). A good agreement of the papilla profile between the LF system and OCT was shown (Fig. 15).

A linear relationship between the physical papilla depth of the eye model and the measured depth was demonstrated (Fig. 9), as was the relationship between OCT measurements and LF depth measurements (Fig. 13). Here an offset in the linear approximation of these measurements was found in contrast to the linear approximation of the eye-model measurements. We suspect that the reason for this is the unknown calibration reference of the OCT depth measurement.

However, only six subjects were examined in this study, and therefore, no statistical statement is possible. We consider our results to be preliminary due to the sparse data situation. It was shown that a depth measurement *in vivo* is possible because the depth extension of the examined papillae with suspected glaucoma was large enough to show the expected effect. Since all subjects with suspected glaucoma show neither visual field defects nor other abnormal glaucoma parameters, it can be assumed that the respective glaucoma development is still in its early stages. Thus the LF system has the potential for early diagnosis of suspected glaucoma.

The LF fundus camera is not calibrated metrically; the depth specifications only correspond to a virtual estimation in v-mm. To perform such a calibration, a specially developed eye model is necessary, including a measurement scale, as is taking the mean spherical aberrations of the human eye into account. Alternatively, a correspondingly adapted calibration procedure is required.^27^ This calibration is then ultimately valid for this system, including software with its specific settings.

Using the calculated system magnification as a preliminary calibration, the LF measurements were corrected. Results showed only small measurement differences from the OCT data at deep papillae. One reason for this may be the uncertainty of finding the same measurement position in both systems. In addition, it is possible that the selected OCT scan does not exactly match the depth section of the LF system.

(2) On which structures is the depth estimated?

Since the depth estimation algorithm needs a certain minimal image contrast in each microlens image, and depth estimation only works in image areas with structure, the components must be small enough to be imaged with at least two microlenses. The structures for which the depth at the retina can be estimated are mainly the vessels, papilla border, and structures within the papilla. This finding is similar to the results of Palmer et al.^11^ All other regions are too homogenous.

(3) What wavelength produces the most depth information?

The highest vessel contrast occurred with green light (Fig. 11, 520 nm), which corresponds with findings from other publications.^28^^,^^29^ Hence, most depth estimation points were found at that wavelength (Fig. 12). Two percent more measurement points were found in subject S4 at a wavelength of 600 nm. A non-optimal focus of the LF fundus camera regarding the measurement range of the LF imager led to more artifacts and thus to an image structure that was falsely detected for depth estimation.

(4) Are structures at deeper layers, such as the choroidea, measurable?

Longer wavelengths penetrate deeper into the fundus^30^^,^^31^ so that deeper layers, especially the choroidea, become more prominent in the image, but the contrast is not sufficient for depth estimations. Marshall et al.^23^ showed, theoretically, that the photon direction order is destroyed by scattering in deeper layers. Due to the higher reflectivity of blood in the red spectrum, retinal vessel contrast is reduced, so depth estimation is unlikely to work. With increasing wavelength, a contrasting reversal can be observed. This is already visible at 650 nm (Figs. 10 and 12). Vessels appear bright on a dark background. With the use of infrared wavelengths, it might also be possible to make choroidal structures so clearly visible that depth could be estimated there.

(5) What kind of image artifacts occur?

Concerning image artifacts, LF technology is demanding for imaging optics. The f-number of the optical system should fit the f-number of the microlens array for the highest depth resolution. The f-number of the fundus camera used here corresponded to a pupil diameter of about 3 mm, resulting in an f-number of about 5.6. Despite that mismatched imaging, refocusing and depth estimations were possible. Fitting the f-numbers with a new optical design can increase the depth resolution. By increasing the aperture corresponding to a larger pupil diameter, the separation of illumination and imaging paths becomes challenging. Back reflections from the cornea and lens result in artifacts in many microlenses overlaying image information that could be used for depth estimation.

The problems of typical fundus photography, such as wavefront errors, for example, due to tear film breakup, result in blurred but mostly usable images. Reflections and scatter from the anterior segment of the eye mainly reduce the image contrast. With imaging through a microlens array, the artifacts are imaged in many subimages with low spatial frequency and superimposed with the subsequent image reconstruction. Since the artifacts of the subimages are not located in a fundus conjugate plane but typically in front of it and partly outside the measuring range of the sensor, these artifact images are superposed or reconstructed incorrectly or only insufficiently, i.e., a two-dimensional artifact is only reproduced selectively. This then corresponds approximately to the grid of the microlens array. In LF fundus photography, those artifacts in many microlenses and in reconstruction defects make image regions or the whole image useless for further analysis or diagnosis (Fig. 16). Image acquisition is therefore more challenging than in common fundus photography. The artifacts shown in Fig. 16 have strong frequency components because of the multiple imaging of scattering and reflection by the microlens array. Possibilities for hardware artifact reduction can be an antireflective coating of the microlens array or the use of polarization effects at the cornea. On the software side, such artifacts can only be reduced to a limited extent by frequency-dependent filtering since image structures such as vessels lie in a similar frequency spectrum. The artifacts can also be reduced by reflex detection in the raw data image, whereby corresponding areas are omitted for image reconstruction. Although the fundus surface is spherical with a radius of about 10 mm, the fundus is imaged flat, and the roundness is not measurable. The reason for this is the imaging optics of the fundus camera, which are designed to image the curved fundus surface onto a flat sensor. Since the optics design was created with a standard eye model, only deviations from the spherical shape of the underlying model can be imaged three-dimensionally.^32^ These effects are corrected in 3D-OCT applications, which is also possible for retinal LF imaging with an appropriate eye model.

The eye model used here was designed with regard to the following aspects: a 1:1 scale to the human eye; modeling of the back reflections, especially of the cornea; and interchangeable fundus models with different papilla excavations. Many eye models imitating the structure of the human eye and modeling individual aspects of optical imaging have already been presented in the literature.^33^^–^^38^ They all have a certain optical complexity in common, but this was not the main focus here. With the demand for interchangeable fundus models, a relatively simple eye model was developed that is easy to handle and provides good optical results in small image fields (30 deg). Results of the eye model study support the findings of the subject study.

Artifacts in the depth map are vessels at different depths. The retinal vessels should be imaged on one level; therefore, they have almost no depth differences. In the images of subject S4 at 520 nm (Fig. 12) especially, these artifacts become obvious. Horizontal vessels are mapped yellow and thus seem to lie further in the foreground than the vertical green mapped vessels. One reason for this may be the subject’s astigmatism of −0.5 dpt in a vertical orientation. Depth estimation of horizontal structures is mainly done by subimages lying next to each other in the vertical direction. The depth of vertical structures is accordingly estimated from horizontally adjacent subimages. However, both subimage orientations have different distortions due to the same directional astigmatism. Therefore, the depths of vertical structures show a difference compared with horizontal structures. Since the papilla depth was determined from horizontal and vertical reference points, this error was suppressed in the evaluation by averaging. However, hardware optics correction is necessary for accurate data correction. Digital correction with a known error is also conceivable. Both approaches require further investigation.

Two-dimensional imaging of an optic disc suspected of glaucoma still poses a challenge for image interpretation. With three-dimensional imaging of the optic disc, suspected glaucoma can be diagnosed more easily and reliably than with two-dimensional imaging. In this context, optic disc depth is only one possible parameter for predicting the current glaucoma status, which can support the clinical diagnosis.^39^^,^^40^ However, looking at the complete shape of the excavation of the papilla, it should be possible to establish more risk parameters, such as the angle of the excavation walls, volumetric parameters, or a cup-to-disk ratio equivalent, for example. The limitations of the introduced and tested combination of the fundus camera and LF imager are the non-perfect aperture matching and a predefined field of view, leading both to a reduced depth resolution. Therefore, a well-balanced optical design and stop design, concerning requirements related to the LF imager and the fundus camera, will be considered more precisely in a future step. With matching apertures and a smaller field of view of 15 deg to 20 deg (large enough to image the optic disc sufficiently well), the depth resolution can be increased significantly. In summary, LF technology has the potential to help diagnose and monitor glaucoma risk at an early stage through a rapid, cost-effective technology, if the LF imager, which is actually the main cost factor of the system, becomes more cost effective.

## 5 Appendix

The Appendix contains Table 1 with the parameters of the Raytrix LF software (RxLive 5.0.046.0, Raytrix GmbH, Kiel, Germany). The used parameter values are listed and a short description of the respective parameter is given.

**Table 1:** Processing and view parameters of the LF software with the used values.

Processing parameter (expert mode)		Explanation
Preprocessing		
Gradation line	Disabled	Disables the change of the gradation line of the image histogram
Denoise	Disabled	Disables the denoise algorithm
Sharpening	Disabled	Disables the sharpening algorithm
ROI	Disabled	Disables the image size reduction by limiting to an ROI
Focus		
Depth blending scale	1.00	Overlay of the depth map in the total focus image
Focus resolution (px)	2012 × 1518	Resolution of the total focus image
Depth estimation		
Depth algorithm	Raycast	Type of triangulation algorithm
Min. depth (%)	0.00	Measuring range limitation
Max. depth (%)	130.00	
Enabled lens types	Near, middle, and far	Microlens type used for triangulation
Min. correlation	0.860	Minimum correlation coefficient for autocorrelation of adjacent image patches, 1 = patches are identical
Min. std deviation	0.006	Minimum standard deviation for autocorrelation of adjacent image patches, used as proxy for contrast/structure, high standard deviation indicates high contrast
Patch diameter (px)	5	Patch diameter used for autocorrelation
Patch stride (px)	1	Spatial resolution of patch matching
Consistency check	Enabled	Enables the data consistence check by reverse calculation
Depth map creation		
Depth map resolution	1006 × 759	Defines the resolution of the depth map
Filling	Enabled	Enables the gap filling algorithms for the depth map
Fill raw	Disabled	Disables the gap filling within the raw image by reverse calculation
Fill algorithm	Standard	Filling algorithm used
Iterations	0	Filling iterations, 0 = no filling, only filter is active
Lookup distance (px)	1	Filling with next pixel
Complete fill	Disabled	Disables the complete filling of gaps in the depth map
Filter	Enabled	Enables the filter within the filling algorithm that deletes inconsistent depths
Bilateral filter	Enabled	Enables the following bilateral filter
Bilateral algorithm	Bilateral2D	Filter algorithm used
Filter radius (px)	10	Filter radius
Edge smoothing factor	0.025	Smoothing factor
View parameter		
Position (mm in global)		
x	0.00	Position of the reference plane
y	0.00	
z	0.00	
Orientation (deg in global)		
Pitch deg	0.00	Orientation of the reference plane
Roll (deg)	0.00	
Yaw (deg)	0.00	
Move (mm in reference)		
Step size (mm)	0.00	Data positioning with respect to the reference plane
x	0.00	
Y	0.00	
z	0.00	
Depth limits (mm in reference)		
Min. depth	0.5	Borders of the colored depth map in Z direction
Max. depth	6.0	
Coloring parameter		
Color map	Perceptually uniform rainbow	Color of the depth map
